# Effect of Mo doping in NiO nanoparticles for structural modification and its efficiency for antioxidant, antibacterial applications

**DOI:** 10.1038/s41598-023-28356-y

**Published:** 2023-01-24

**Authors:** Mir Waqas Alam, Amal BaQais, Tanveer Ahmad Mir, Insha Nahvi, Noushi Zaidi, Amina Yasin

**Affiliations:** 1 Department of Physics, College of Science, King Faisal University, Al-Ahsa, 31982 Saudi Arabia; 2 Department of Chemistry, College of Science, Princess Nourah Bint Abdulrahman University, Riyadh, 11671 Saudi Arabia; 3 Laboratory of Tissue/Organ Bioengineering and BioMEMS, Transplantation Research & Innovation (Dpt)-R, Organ Transplant Centre of Excellence, King Faisal Specialist Hospital and Research Centre, Riyadh, 11211 Saudi Arabia; 4 Department of Basic Sciences, Preparatory Year Deanship, King Faisal University, Al-Ahsa, 31982 Saudi Arabia

**Keywords:** Materials science, Energy science and technology

## Abstract

Novel molybdenum (Mo)-doped nickel oxide (NiO) Nanoparticles (NPs) were synthesized by using a simple sonochemical methodology and the synthesized NPs were investigated for antioxidant, and antibacterial applications. The X-ray diffraction (XRD) analysis revealed that the crystal systems of rhombohedral (21.34 nm) and monoclinic (17.76 nm) were observed for pure NiO and Mo-doped NiO NPs respectively. The scanning electron microscopy (SEM) results show that the pure NiO NPs possess irregular spherical shape with an average particle size of 93.89 nm while the Mo-doped NiO NPs exhibit spherical morphology with an average particle size of 85.48 nm. The ultraviolet–visible (UV–Vis) spectrum further indicated that the pure and Mo-doped NiO NPs exhibited strong absorption band at the wavelengths of 365 and 349 nm, respectively. The free radical scavenging activity of NiO and Mo-doped NiO NPs was also investigated by utilizing several biochemical assays. The Mo-doped NiO NPs showed better antioxidant activity (84.2%) towards ABTS. + at 200 µg/mL in comparison to their pure counterpart which confirmed that not only antioxidant potency of the doped NPs was better than pure NPs but this efficacy was also concentration dependant as well. The NiO and Mo-doped NiO NPs were further evaluated for their antibacterial activity against gram-positive (*Staphylococcus aureus* and *Bacillus subtilis*) and gram-negative (*Pseudomonas aeruginosa* and *Escherichia coli*) bacterial strains. The Mo-doped NiO NPs displayed better antibacterial activity (25 mm) against *E. coli* in comparison to the pure NPs. The synthesized NPs exhibited excellent aptitude for multi-dimensional applications.

## Introduction

The excessive utilization of the noxious chemical substances for the purpose of food preservation have emerged as one of the major concerns for the scientific community in recent times. These chemicals utilize their antimicrobial potency and antioxidant potential in order to perform the food reservation process by modulating the metabolic oxidative and microbial food spoilage problems. The organic foods particularly fruits, vegetables, fish, meat, poultry, and fresh cereal-based products are largely affected by the microbial spoilage (i.e. the degradative reactions performed by bacteria, fungi and algal strains^1^. The onset of the food spoilage is remarked by the pronounced variation observed in the texture, taste, color, and odour of the food. Furthermore, the physical parameters including light, moisture content, temperature, and air flow etc. also modulates the degradation rate of the food by directly impacting the working of the enzymes and proteins (of the microorganism) present in the food. Usually, the favourable the conditions are for the enzymatic degradation, the larger are the number of the oxidative free-radicals produced in the food which results in its spoilage^2^.

The reactive oxidative species (ROS) are extremely unstable species containing an unpaired electron in its atomic orbitals. The presence of the unpaired electron makes these radicals quite reactive and these free-radicals destroy the surrounding food molecules via numerous destructive mechanisms (i.e. via undifferentiated bond breakage of the biomolecules of proteins, carbohydrates, nucleic acids etc.). Even in the absence of the microbes, the oxidative free-radicals are usually generated during the metabolic conversion of the consumed food in the human beings as well^3^. The excessive presence of these free-radicals inside the human cell is quite dangerous as these species constantly interact with the constituents of the human cells and hinders its proper functioning^4^. Recently, the overproduction of these ROS in human body have been heavily linked with the onset of numerous diseases including immunity system failure, neurological diseases, cancer, neuro-degenerative ailments and certain cardiovascular diseases^5^. Similarly, the enhanced aging phenomenon was also found to be related with the adverse oxidative impacts of these ROS^6^. Consequently, it can be observed that the modulation of the proper amount of the ROS in the food and the human body is an essential research domain which should be explored when dealing with food preservation and biomedical applications, respectively^7^.

In case of living organisms, human beings are equipped with natural defence mechanisms to counter the deterioration effects of the oxidative species^8^. For instance, the consumption of meat and plants (particularly plants) allows us to consume natural antioxidant substances that scavenge these ROS from the human body. However, the natural intake of these antioxidant substances is incidental and supplementation in the form of the synthetic antioxidants (including butylated hydroxytoluene (BHT), and butylated hydroxyanisole (BHA)) is required for properly modulating these ROS based oxidative stress^9^. Although BHT and BHA are one of the two most extensively utilized antioxidants, the focus has now been shifted towards the identification or development of the novel antioxidants as the synthetic antioxidants of BHA and BHT have been documented to have carcinogenic issues associated with them^10^. The nanomaterial (NMs), substances having at least one of its dimensions within the nanometers range (i.e. 1–100 nm), are one such material that have been documented to be extensively utilized as the antioxidant substance for controlling the oxidative stress in foods and human beings^11^. The NMs are regarded as the unique substances that exhibit the diverse characteristics (surface, physical, optical, chemical, and electrochemical properties) owing to their extremely small size^12^. The surface area associated with these NMs is exponentially higher in comparison to their bulk counterpart which make these materials excellent substances for surface dependent applications such as water purification applications (including adsorption, catalysis, photocatalysis, etc.), electronics and biomedical applications (antibacterial, antifungal, and anticancer applications, etc.)^13^. The antimicrobial applications are particularly dependent upon the size of the NPs. The smaller the size of the NPs, the easier it is for the NPs to penetrate into the microbial cells and cause lysis. Consequently, it can be said that the features of intensely small size and high surface area of the NPs are exceedingly significant for the numerous applications of the NPs^14^.

The NMs, particularly transition metal oxide nanoparticles (NPs), have been known for numerous applications owing to their antioxidant properties performed via two mechanisms: i.e. either by quenching of the radicals via chain breaking processes or by supressing the production of the oxidative radical promoters via chelation processes^15^. Ge et al.^16^ presented an interesting fact that among both the metal and metal oxide NPs, the metal oxide NPs exhibited better antioxidant potential owing to the involvement of the oxygen atom in their structures. Presence of the oxygen atom not only enhances the chelation capabilities of the metal oxide NPs (by developing the electrostatic interactions with the radicals that are missing in neutral metal NPs) but it also increases the quenching potential of the synthesized NPs (owing to the presence of the different oxidation states of the metal ions involved in the numerous oxides of the transition metal oxide NPs)^16^. Muthuvel et al.^17^ synthesized titanium oxide NPs and utilized the synthesized assembly for photocatalytic, antioxidant and antibacterial infections. Haq et al.^18^ synthesized nickel oxide (NiO) NPs by utilizing the green synthesis methodology for antimicrobial and antioxidant properties. Keeping the above discussed points in mind, it can be inferred that the metal oxides are one of the most prominent NMs that are explored for investigating the antioxidants and antimicrobial applications.

For this particular study, we have focused on developing the doped NiO NMs. The NiO is an exceptional transition metal oxide known for its insulation nature (i.e. low conductivity values at room temperature) and is widely used in the fields of the electrochromic materials, supercapacitors, gas sensors and battery manufacturing^19^. This p-type semiconductor material also exhibits the bandgap value of 3.51 eV making these materials quite suitable for catalytic applications^20^. The NiO NPs are modified in numerous ways to achieve better results such as formation of nanocomposites^19,20^, surface modifications^21^, and doping process^22^. All these processes are designed in such a way that the electrical, optical, or structural characteristics of the NiO NPs are improved for the specific applications. The process of doping includes the introduction of the specific transition metals/impurities within the crystal system of the NMs in order to modulate the structural, optical, and electrical properties of the parent NMs. Achieving the doping is quite difficult task as the crystal lattice system of the NMs has to remain intact and only one or more atoms present at the specific points in the crystal unit have to be replaced by the dopant atom^22^. Literature survey associated with the intercalation studies of NiO NPs reveal that mostly copper (Cu), cobalt (Co), magnesium (Mg), and potassium (K) are utilized for the doping of the NiO NMs. Ethiraj et al.^23^ synthesized Cu doped NiO NPs and indicated that the optical property of the bandgap increased from 3.26 to 3.67 eV by increasing the dopant concentration from 0 to 4% respectively. Lee et al^24^ also synthesized Co doped NiO NPs and documented that the electrical conductivity and interfacial characteristics of the NiO NPs were enhanced by the doping process. All these case studies indicated that doping of the NiO NPs is an excellent technique for improving the properties of the NPs.

Here, the pure NiO NPs and molybdenum (Mo) doped NiO NPs were synthesized by using ultrasonic assisted wet chemical synthesis. The selection of Mo was done on the basis of its natural affinity to act as a cofactor for numerous enzymes in biological systems. As the main focus of our study was to synthesized doped NiO NMs and employ it for the antibacterial and antioxidant applications, the biocompatible dopant of Mo was selected^22^. Apart from these applications, the authors made an attempt to explain the mechanism involved in the antibacterial activity of the NPs which adds on to the novelty of this study. The mechanism studies explaining the process of the bactericidal activities of the NPs are rarely explored in the academic literature. Authors believe that this study will provide significant insights in understanding the mechanisms involved in the interaction of pure and doped NMs with the bacterial cell.

## Results and discussion

### X-ray diffraction (XRD) analysis

**Figure 1:**
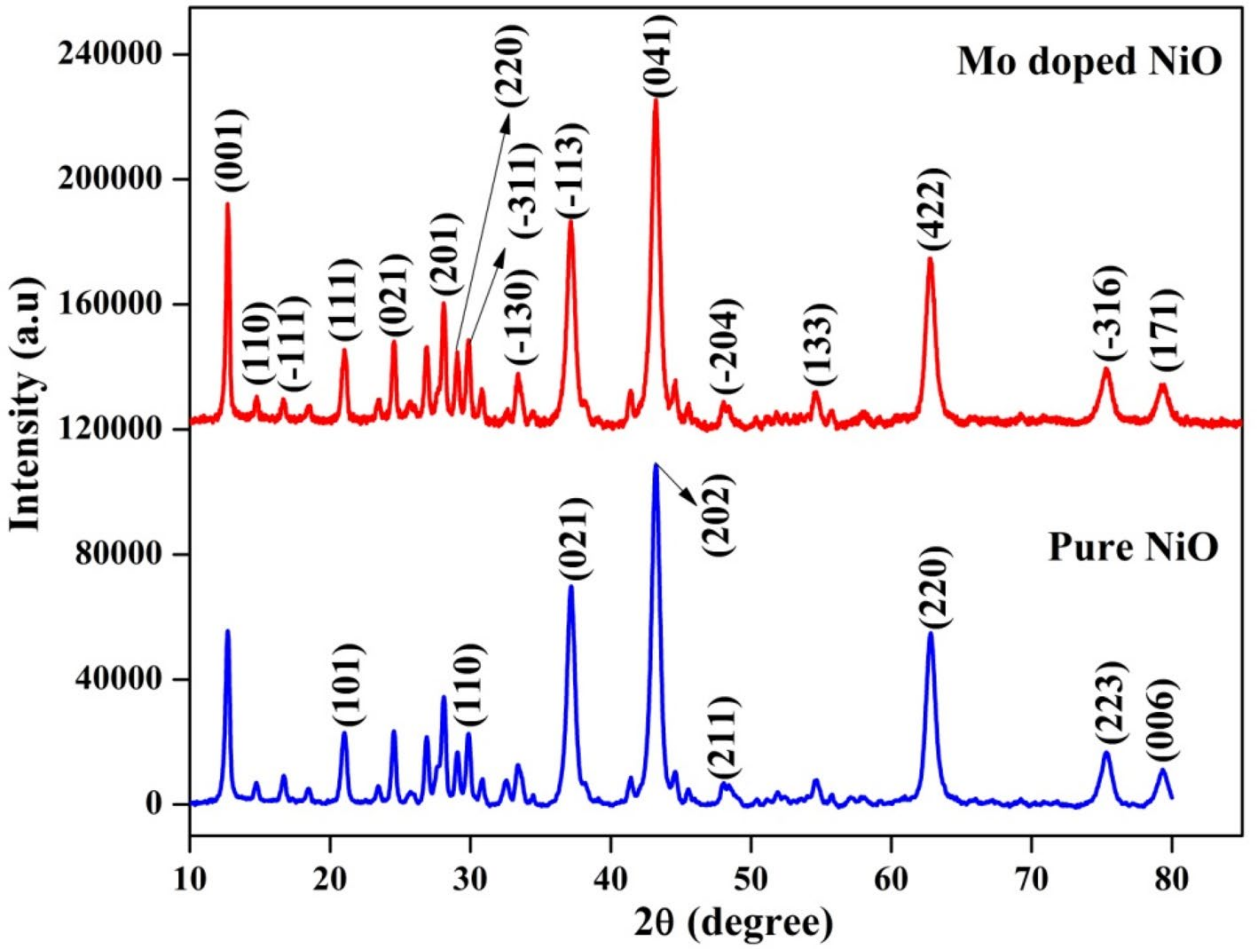
XRD analysis of NiO and Mo doped NiO NPs.

The average crystallite size and crystal phase associated with the synthesized NP were investigated by using XRD analysis. Figure 1 depicts the XRD pattern of pure NiO and Mo doped NiO NPs. The crystal phases of rhombohedral (JCPDS card No. 89-3080) and monoclinic (JCPDS card No. 86-0361) were observed for pure NiO and Mo doped NiO NPs, respectively. For pure NiO NPs, it was observed that the 2θ values of 21.16°, 30.78°, 37.19°, 43.18°, 48.20° 62.84°, 75.39°, and 79.30° corresponded to the respective diffraction planes of (101), (110), (021), (202), (211), (220), (223), and (006). In case of Mo doped NiO NPs, the 2θ values of 12.73°, 14.74°, 16.70°, 21.01°, 24.40°, 28.06°, 28.97°, 29.94°, 37.19°, 43.29°, 47.92°, 54.63°, 62.78°, 75.26°, and 79.30° corresponding to the diffraction planes of (001), (110), (-111), (111), (021), (201), (220), (-311), (-113), (041), (-204), (133), (422), (-316), and (171) respectively were observed. The slight changes observed in the 2θ values of Mo doped NiO NPs in comparison to the 2θ values of pure NiO NPs is indicative of the fact that the parent structure of the NiO NPs remains the same and Mo is only incorporated as an impurity in the system^25^. For instance, the slight difference of 0.11° was observed at the major diffraction peak of NiO NPs (at 43.18°) which was shifted at the 2θ value of 43.29° in the case of Mo doped NiO NPs. The average crystallite size of the NPs was calculated by using the Debye Scherrer formula. The average crystallite size of pure NiO and Mo doped NiO was found to be 21.34 nm and 17.76 nm respectively. Panigrahi et al.^26^ has investigated the effect of Mg and Zn doping on NiO nanoparticles by changing the ratio of dopant and the pure NiO exhibited cubic phase. According to the Panigrahi’s report, the parameters of both bond length and lattice parameters exhibited increase with the increase in the ratio of metal dopant^26–28^. According to the above mentioned literature, the main dopant of Mo is responsible for the crystal structure observed in case of the doped NiO NPs.

### Scanning Electron Microscope (SEM)-energy dispersive X-ray analysis (EDAX) analysis

**Figure 2:**
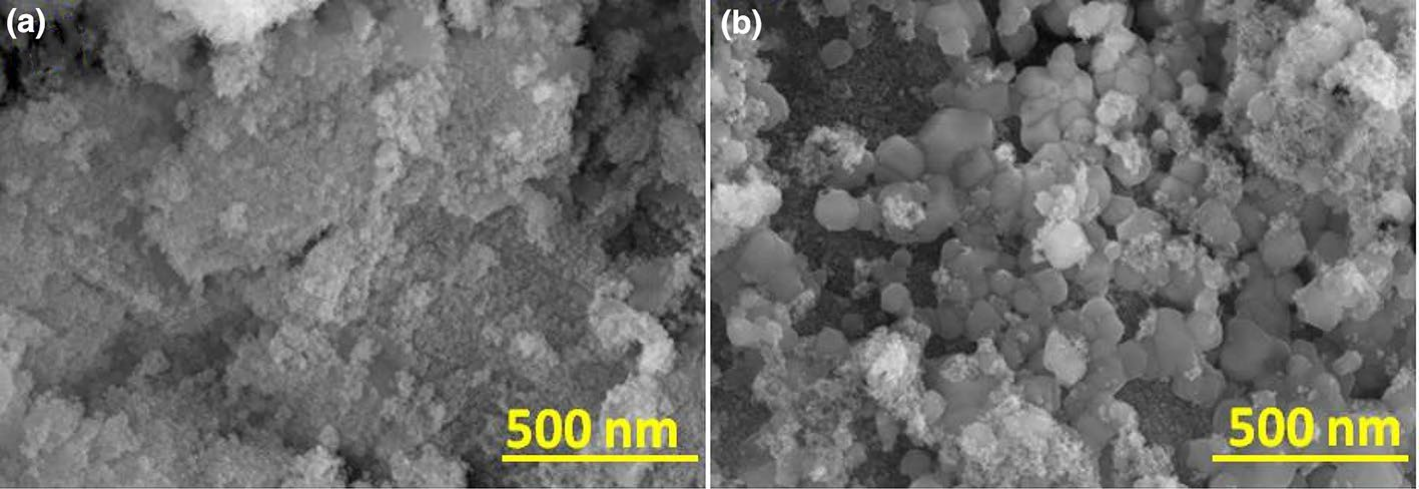
SEM analysis of (**a**) NiO and (**b**) Mo doped NiO NPs.

Figure 2 depicts the SEM analysis of pure NiO and Mo doped NiO NPs. From the analysis, it was observed that both the pure NiO and Mo doped NiO NPs possessed irregular spherical shaped NPs with the prominent aggregation observed in both cases. The aggregation of particles is common for NMs owing to the involvement of strong interparticle contact-induced interactions generated in the NPs because of the high surface energy of the NPs^27^. The average particle size of pure NiO and Mo doped NiO NPs (inferred from ImageJ software) was found to be approximately 93.89 and 85.48 nm respectively. Ma et al.^29^ has synthesized NiMoO_4_ nanoparticles via hydrothermal route and nano needle structure was observed for NiMoO_4_ nanoparticles.

**Figure 3:**
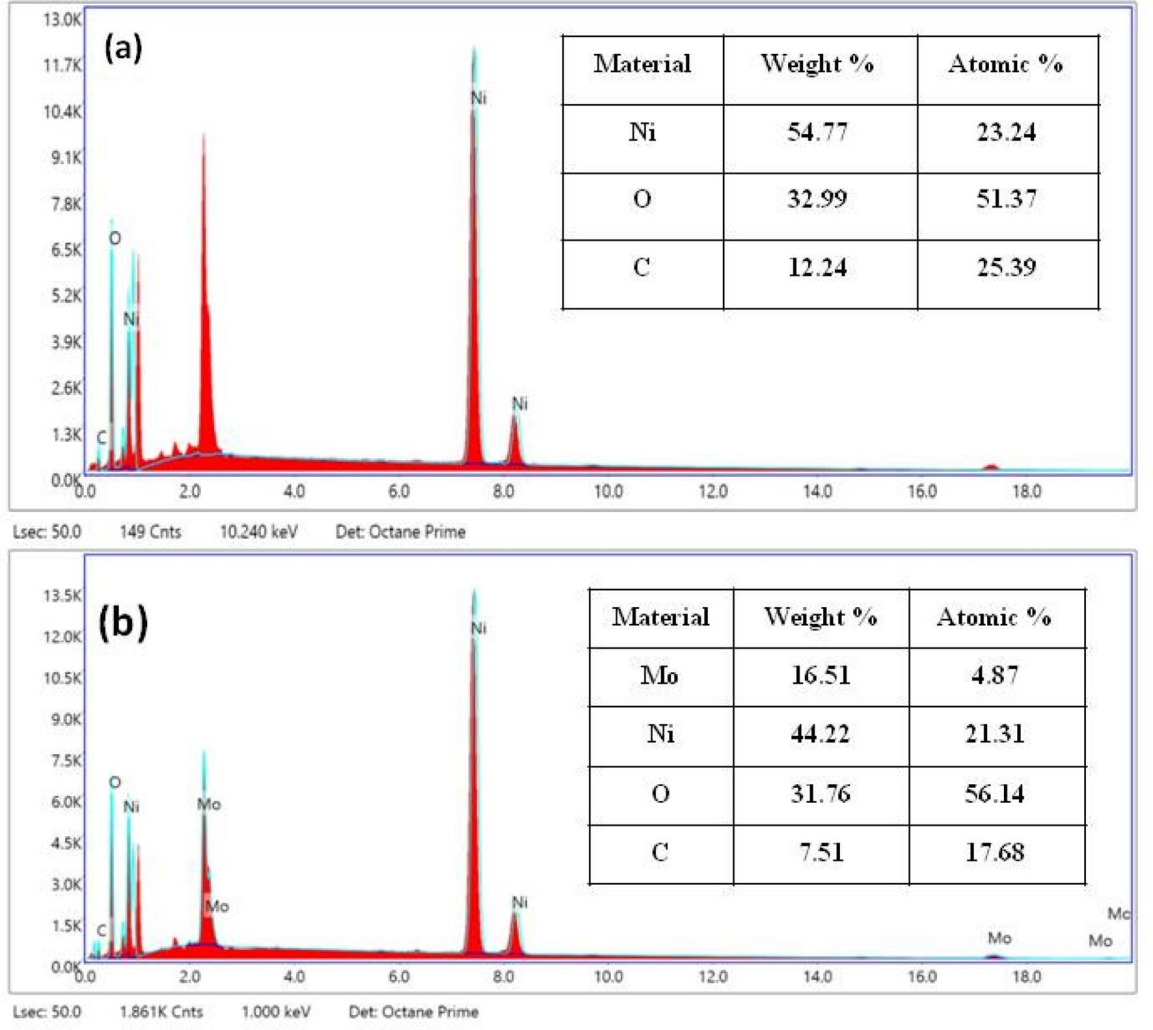
EDAX analysis of (**a**) NiO and (**b**) Mo doped NiO NPs.

The elements present in pure NiO and Mo doped NiO NPs were identified by using EDAX analysis (Fig. 3). In pure NiO NPs, the weight percentages of Ni, O and C were found to be 54.77%, 32.99% and 12.24% respectively while the atomic percentages of Ni, O and C were found to be 23.24%, 51.37% and 25.39% respectively. In Mo doped NiO NPs, the weight percentages of Mo, Ni, O and C were found to be 16.51%, 44.22%, 31.76%, and 7.51% respectively while the atomic percentages of Mo, Ni, O and C were found to be 4.87%, 21.31%, 56.14%, and 17.68% respectively.

### High resolution-transmission electron microscopy (HR-TEM) analysis

**Figure 4:**
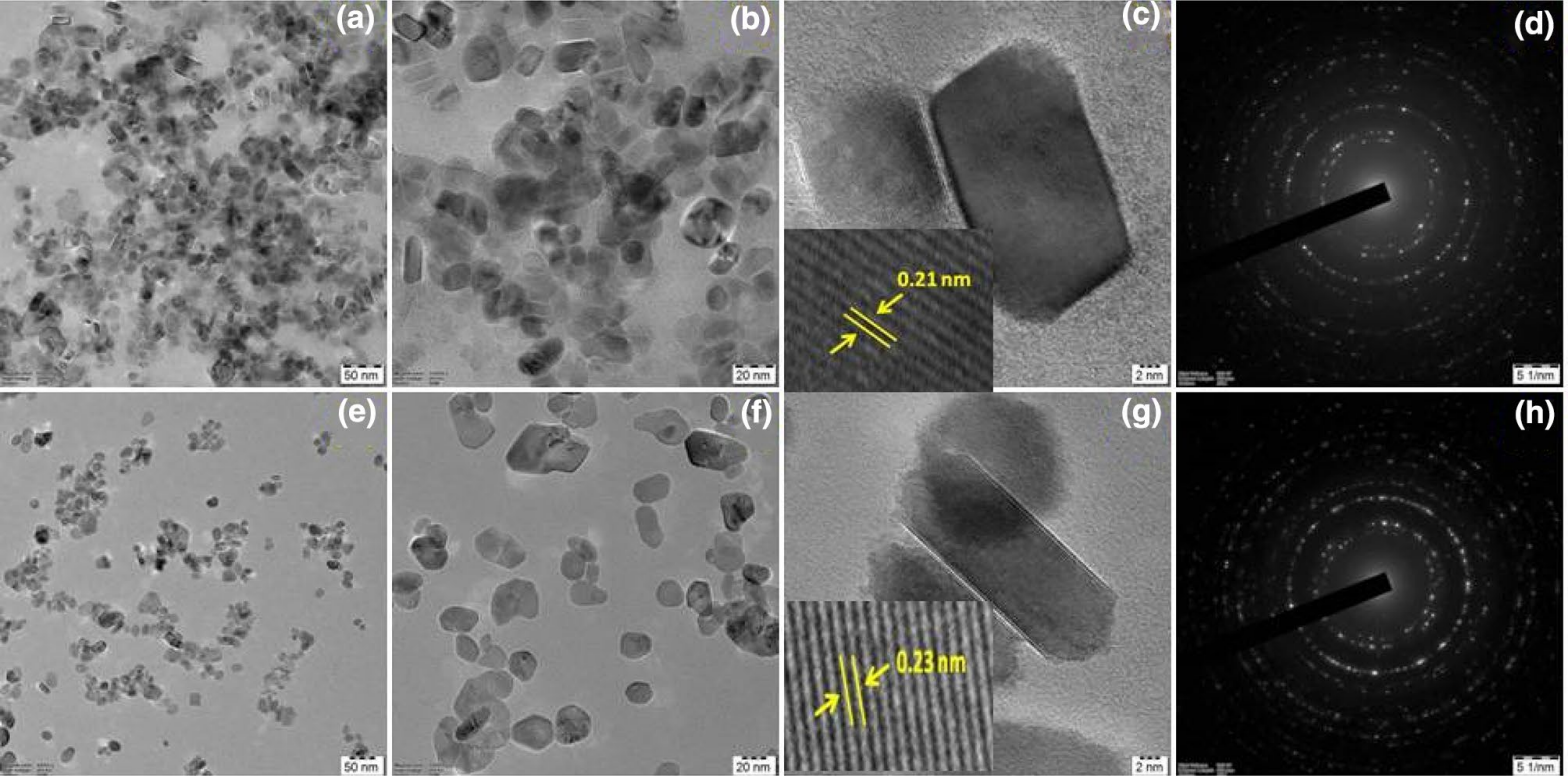
HR-TEM analysis of (**a**–**c**) NiO NPs and (**e**–**g**) Mo doped NiO NPs; SAED pattern of (**d**) NiO NPs and (**h**) Mo doped NiO NPs.

The HR-TEM micrographs of pure NiO and Mo doped NiO NPs at three different magnifications (50 nm, 20 nm, and 02 nm) are shown in Fig. 4 (a-h). From the micrograph, it was observed that the pure NiO and Mo doped NiO NPs exhibited rectangular and rod like morphology respectively. The particle size of the NPs observed via HR-TEM was found to be higher than the XRD which may be due to the merging of particles or the fact that XRD is an estimation technique. Comparatively, the HR-TEM should be considered more reliable in comparison to the XRD analysis. The average particle size of pure NiO and Mo doped NiO NPs were measured to be 93 ± 4 nm and 85 ± 3 nm respectively. A lattice fringe with the interplanar spacing of 0.21 nm corresponding to the diffraction plane of (202) was observed for pure NiO NPs while the interplanar spacing of 0.23 nm corresponding to the diffraction plane of (041) was observed for Mo doped NiO NPs. The selected area electron diffraction (SAED) model illustrated bright rings that proved the superior orientation of the nanocrystals. The SAED pattern contained many rings with different radii which showed that the pure NiO and Mo doped NiO NPs were found to be crystalline in nature. The diffraction rings corresponded quite well with the XRD results. The rings (from the inside to outside) were indexed to be (101), (110), (021), (202), (211), (220), (223), (006) for NiO NPs and (001), (110), (− 111), (111), (021), (201), (220), (− 311), (− 113), (041), (− 204), (133), (422), (− 316), (171) for the Mo doped NiO NPs.

### Ultraviolet visible spectroscopy (UV–Vis)

**Figure 5:**
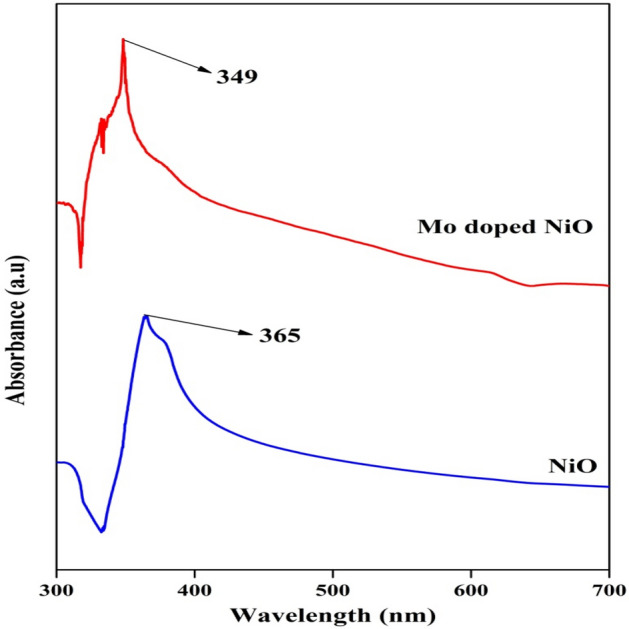
UV–Vis analysis of NiO NPs and Mo doped NiO NPs.

The optical properties including band gap values of the NPs were calculated by using UV–Vis analysis as indicated in Fig. 5. The UV–Vis analysis of pure NiO and Mo doped NiO NPs were recorded in the range of 300–700 nm. The pure NiO and Mo doped NiO NPs showed the absorption maximum in the the visible region at the wavelengths of 365 nm and 349 nm respectively. Furthermore, it should be noted that the difference in the UV–VIS peak is more than 10 nm indicating that the size difference of the pure NiO NPs and Mo doped NiO NPs is quite significant which resulted in the observance of this shift in the UV–VIS spectrum. The doped NiO NPs exhibited the smaller size in comparison to the pure structures indicating that the doped structures should express better antioxidant and antibacterial applications than pure NPs. Riaz et al.^30^ synthesized NiO NPs using *Syzygium cumini* leaves extract and the absorbance value was observed in the range of 290–350 nm. The strong absorption peak associated with NiO NPs was observed at 320 nm which corresponded quite well with our findings. The band gap (E_g_) of NiO and Mo doped NiO was calculated using following formula.

$${\text{E}}_{{\text{g}}} = { 124}0/\lambda$$

According to this, the NiO NPs and Mo doped NiO NPs exhibited the E_g_ values of 3.39 eV and 3.55 eV, respectively.

### Fourier transform infrared spectroscopy (FT-IR)

**Figure 6:**
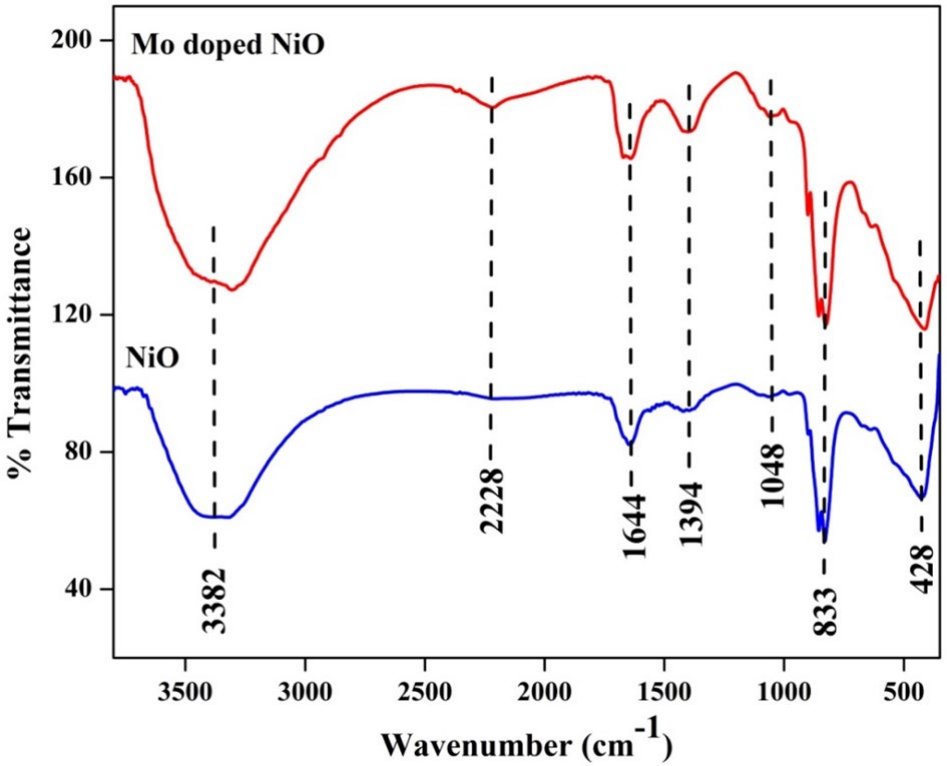
FT-IR analysis of NiO and Mo doped NiO NPs.

The various stretching and bending vibrations of different functional groups present in the synthesized nanoparticles were identified by using FT-IR analysis. The broad peak appeared at 3382 cm^−1^ corresponded to the –OH stretching band from water molecules. The strong absorption band appeared at 1644 cm^-1^ was attributed to the bending vibrations of –OH group in water molecule. The peaks appeared at 1048 cm^-1^ and 1394 cm^-1^ were attributed to the C–O stretching vibration of alcohol and bending vibrations of O–H groups respectively. The peak appeared at 2228 cm^-1^ was attributed to the existence of C = C stretching of alkyne molecules while the peaks observed at 428 cm^-1^ and 833 cm^-1^ were attributed to the bond between metal and oxygen (Ni–O)^31^. The minor peak appeared at 1011 cm^-1^ is indicative of the successful doping of Mo into the NiO crystal lattice^32^. Figure 6 depicts the FT-IR analysis of NiO and Mo doped NiO nanoparticles.

### Antioxidant potential of NiO NPs and Mo doped NiO NPs

**Figure 7:**
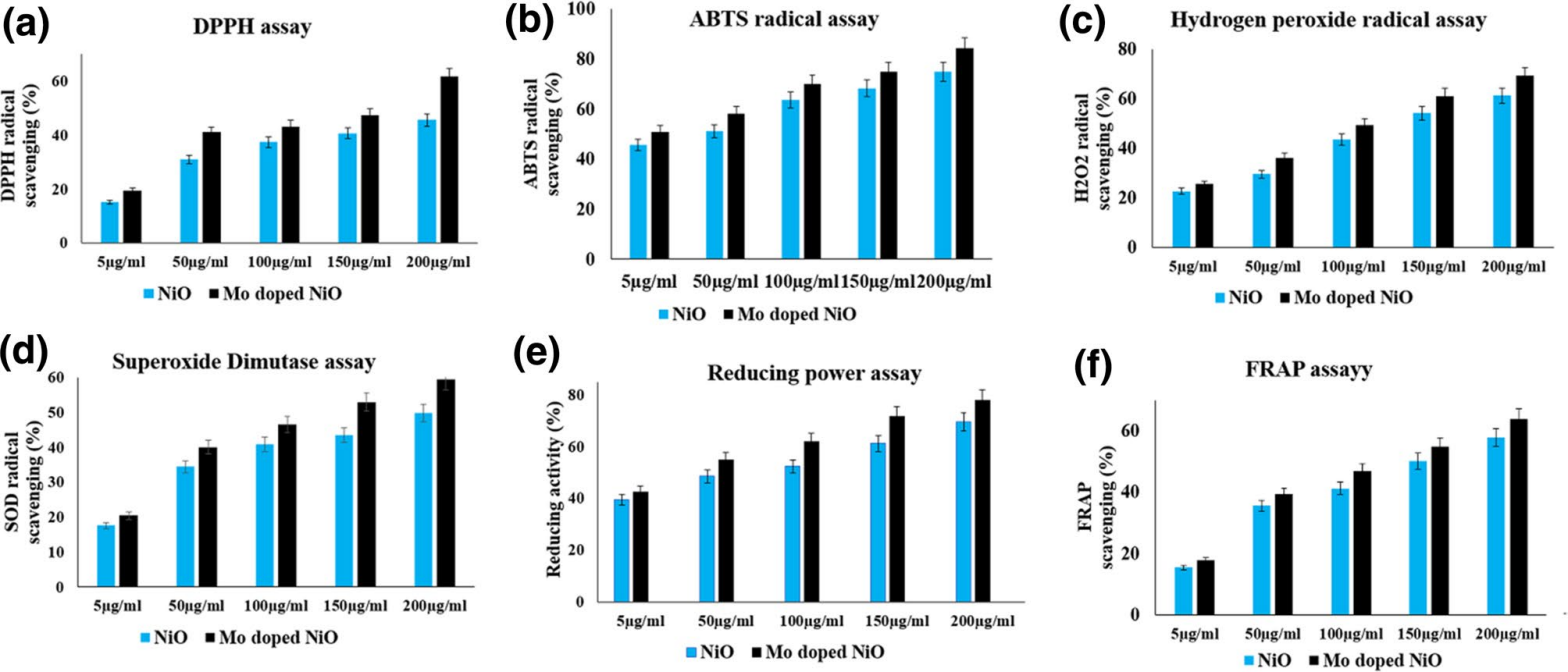
Antioxidant activity of NiO NPs and Mo doped NiO NPs on scavenging the free radicals via biochemical assays of (**a**) DPPH assay,(**b**) ABTS radical assay, (**c**) H_2_O_2_ radical assay, (**d**) SOD assay, (**e**) reducing power assay, and (**f**) FRAP assay.

The antioxidant potential of NiO NPs and Mo doped NiO NPs were assessed by using the in vitro antioxidant efficacy assays. To effectively assess the antioxidant activity, a number of biochemical methods including 2,2-diphenyl-1-picrylhydrazyl (DPPH) assay, 2,2′-azinobis-(3-ethylbenzothiazoline-6-sulphonate) (ABTS^**.**+^) scavenging assay, ferric reducing antioxidant power (FRAP) assay, hydrogen peroxide (H_2_O_2_) assay, reducing power assay, and superoxide dismutase (SOD) assay methods were used for this purpose. The antioxidant activities of NiO NPs and Mo doped NiO NPs are depicted in the Fig. 7. The NiO NPs and Mo doped NiO NPs both exhibited the concentration-dependent DPPH activity. The Mo doped NiO NPs and NiO NPs both successfully scavenge the DPPH radical ions with the respective inhibition percentage of 61.6% and 45.4% at the higher concentrations of 200 µg/mL^33^. The Mo doped NiO exhibited greater DPPH activity than the NiO NPs. In a similar manner, the ABTS^**.**+^ scavenging assays also revealed the same results with the Mo doped NiO NPs exhibiting the maximum inhibition efficacy of 84.2% rather than the 74.8% observed in case of NiO NPs for the concentration of 200 g/mL. At a concentration of 200 µg/mL, the NiO NPs and Mo doped NiO NPs exhibited the radical scavenging activities of 61.2% and 69.2%, respectively for the hydrogen peroxide radicals. The reducing power assay was also performed for the Mo doped NiO NPs and NiO NPs. The doped NPs exhibited better reduction results of 78% in comparison to the pure NPs (69.5%). In case of superoxide scavenging assay, the Mo doped NiO NPs (59.4%) exhibited better efficacy than NiO NPs (49.9%) for scavenging superoxide radical ions. Increases in the concentration of prepared NiO NPs and Mo doped NiO NPs (5 µg/mL, 50 µg/mL, 100 µg/mL, 150 µg/mL, and 200 µg/mL) resulted in an increase in the free radical scavenging activity. Our findings revealed that the Mo doped NiO exhibited greater antioxidant activity than the NiO NPs^32^.

### Antibacterial potential of NiO NPs and Mo doped NiO NPs

**Figure 8:**
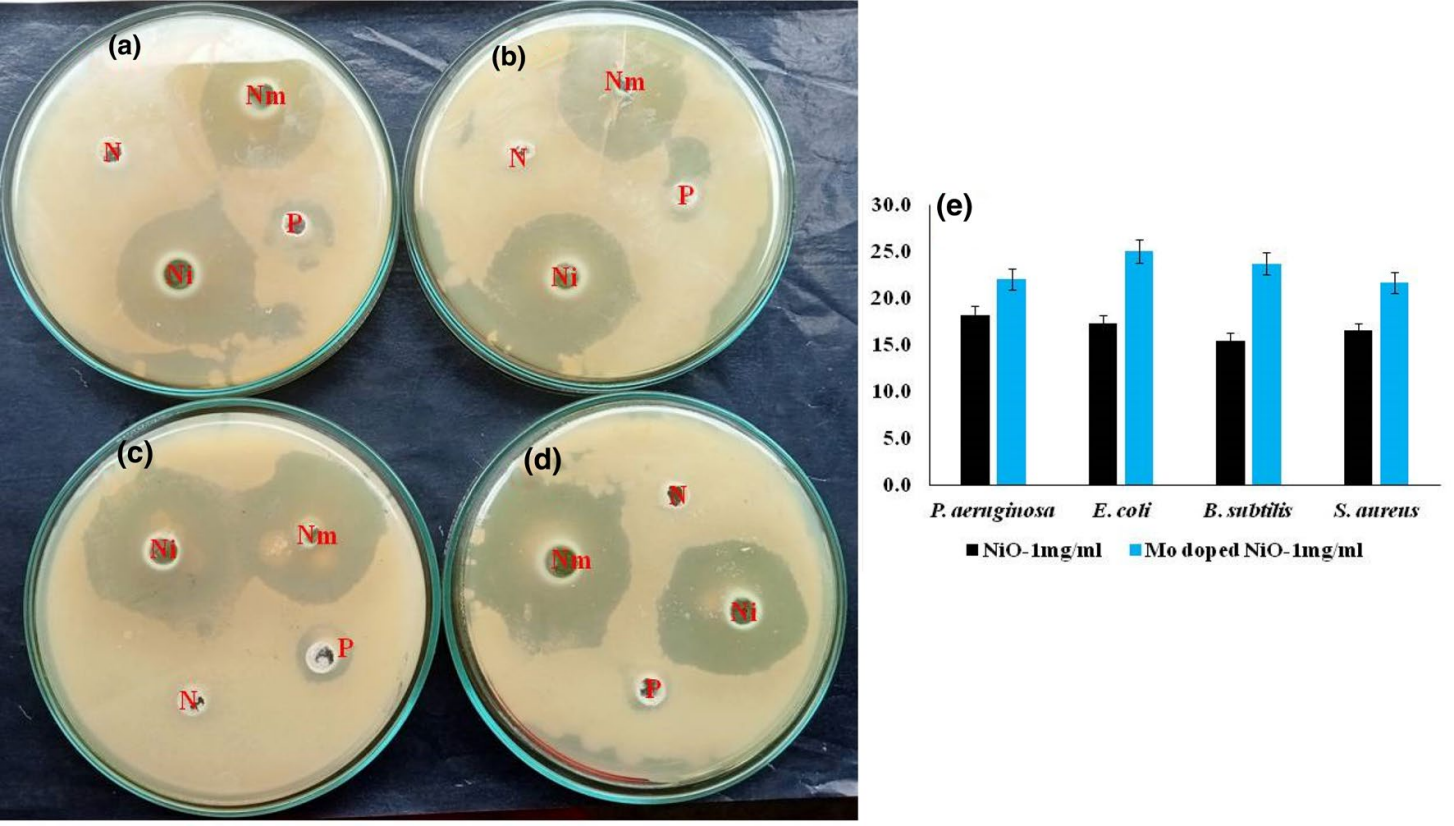
Antibacterial efficacy of Ni and Mo doped NiO against (**a**) *P. aeruginosa*, (**b**) *E. coli,* (**c**) *S. aureus,* and (**d**) *B. subtilis*. (**e**) Comparative bar graph.

NiO NPs have been previously documented to possess antimicrobial potential, however, the mechanism associated with the antibacterial activity of NiO NPs and Mo-doped NiO NPs is not yet reported in the previous studies^34^. In the present study, the potent antibacterial activities of NiO NPs and Mo doped NiO NPs along with their antibacterial mechanism are studied. The bactericidal activity of NiO and Mo doped NiO NPs against the gram-positive strains of *Bacillus subtilis* (*B. subtilis*) and *Staphylococcus aureus* (*S. aureus*) and gram-negative strains of *Pseudomonas aeruginosa* (*P. aeruginosa*) and *Escherichia coli* (*E. coli*). The study revealed that NiO and Mo doped NiO have potent antibacterial properties as indicated in Fig. 8(a–d). The NiO NPs (1 mg/mL) exposed *S. aureus* and *B. subtilis*, *P. aeruginosa* and *E. coli* showed inhibitory zone around 16.5 mm, 15.4 mm, 18.2 mm and 17.2 mm respectively. It was observed that the strains were more severely affected owing to the presence of the doped NPs owing to the fact that the size of these NPs were smaller in comparison to the pure NPs according to HR-TEM results (as indicated in Fig. 8(e)). The smaller size enhances the lysis capabilities by allowing it to easily penetrate into the bacterial cell. The *S. aureus, B. subtilis*, *P. aeruginosa* and *E. coli* were more susceptible to Mo doped NiO NPs (1 mg/mL) and showed an inhibitory zone around 21.6 mm, 23.6 mm, 22 mm and 25 mm respectively. The antibacterial performance of the NiO NPs and Mo doped NiO NPs is typically influenced by the changes in particle size, precursor concentration, pH of the synthesized solution, and surface flaws of the synthesised particles^35^.

**Figure 9:**
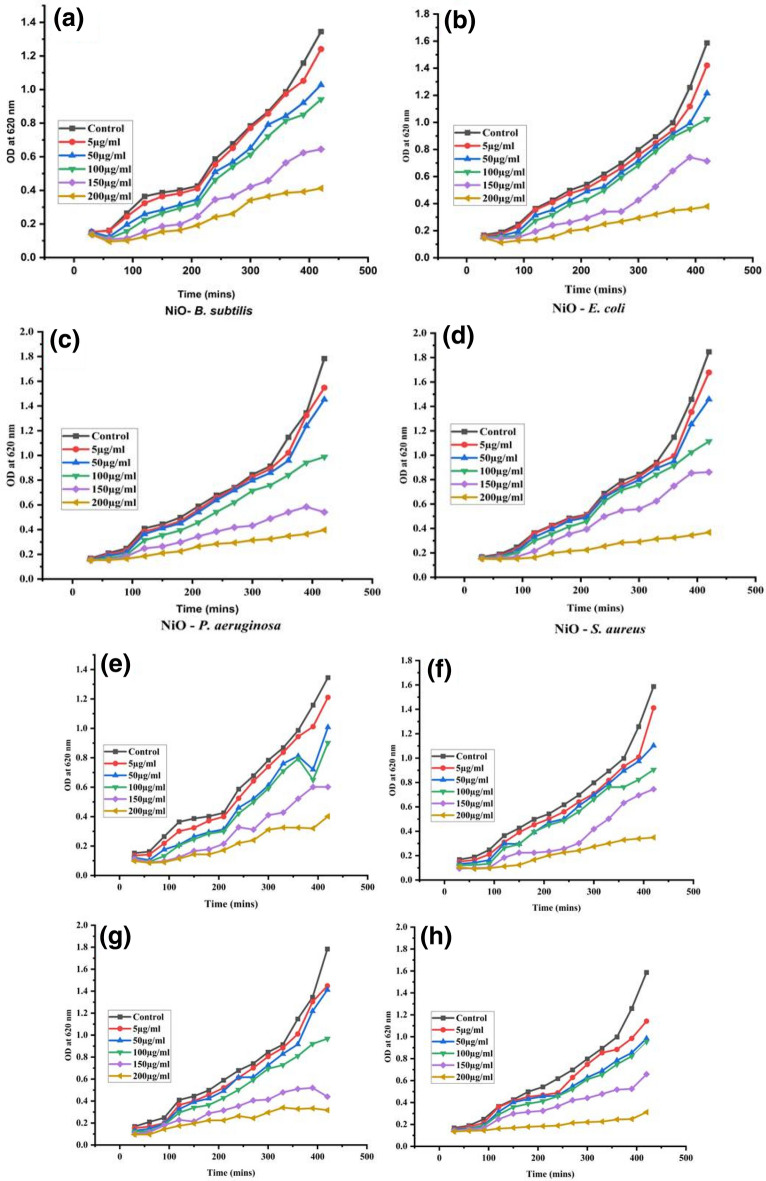
Effect of NiO NPs (**a**–**d**) and Mo doped NiO (**e**–**h**) on growth kinetics of *B. subtilis*, *E. coli*, *P. aeruginosa* and *S. aureus* respectively.

The growth kinetics of *B. subtilis, E. coli, P. aeruginosa* and *S. aureus* were also examined as part of the antibacterial investigation of NiO and Mo doped NiO NPs. The increases in the concentration of NiO and Mo doped NiO NPs (5 µg/mL, 50 µg/mL, 100 µg/mL, 150 µg/mL, and 200 µg/mL) resulted in the increased inhibition of the bacterial growth. The growth of bacteria is limited by NiO NPs, as seen in Fig. 9 (a–d). When exposed to Mo doped NiO, the viability of *B. subtilis, E. coli, P. aeruginosa* and *S. aureus* is inhibited more severely as compared to pure NiO NPs .

The minimal inhibitory concentration (MIC) of NiO NPs and Mo doped NiO NPs required to prevent the visible bacterial growth is determined as well for further studies. The Mo doped NiO NPs exhibited MICs within a range of 3 µg/mL against *B. subtilis*, *S. aureus*, *E. coli*, and *P. aeruginosa*, respectively as shown in Fig. 9 (E–H). Next, we studied the minimum bactericidal concentration (MBC) of NiO NPs and Mo doped NiO NPs. As shown in Table 1, the growth inhibition potential of NiO NPs and Mo doped NiO NPs against bacterial pathogens was observed at a concentration of 4 µg/mL. In comparison to the NiO NPs, the Mo doped NiO NPs effectively inhibited the multiplication of the bacteria tested. This is ascribed to the fact that the Mo as well as the NiO NPs both synergistically contributed to reduce the bacterial viability in case of doped structures^36^. The NiO NPs and Mo doped NiO NPs both showed high antibacterial activity in our study.

**Table 1:** MIC and MBC of NiO NPs and Mo doped NiO. Data presented are average values of three replicates.

Materials	*B. subtilis*		*S. aureus*		*E. coli*		*P. aeruginosa*	
	MIC	MBC	MIC	MBC	MIC	MBC	MIC	MBC
NiO	4	4	4	5	4	4	4	5
Mo doped NiO	3	3	3	4	3	3	3	4

### Antibacterial mechanism of NiO and Mo doped NiO

**Figure 10:**
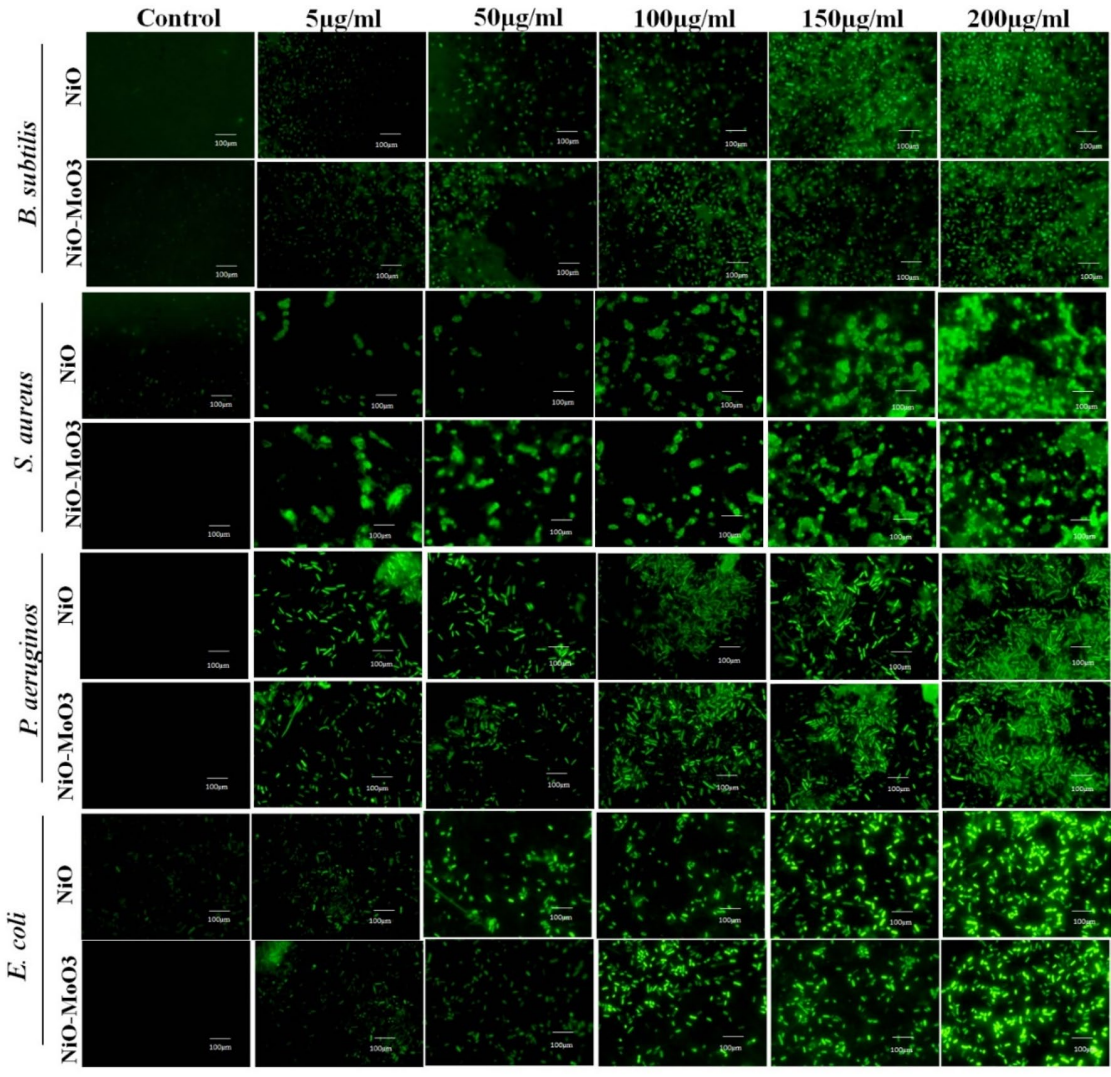
The NiO NPs and Mo doped NiO NPs induced ROS production in bacterial cells *B. subtilis*, *S. aureus* gram-positive *P. aeruginosa*, and *E. coli* in a concentration dependent fashion.

The antibacterial mechanism of NiO NPs and Mo-doped NiO NPs was studied through ROS, deoxyribose nucleic acid (DNA) damage and ROS associated peroxidase (POD), superoxide dismutase (SOD), and malondialdehyde (MDA) activity. We further tested the effect of NiO and Mo doped NiO NPs on the production of ROS in bacteria using H_2_DCF-DA (fluorescent probe). The ROS generation in the bacterial cells were increased when the concentration of NiO NPs and Mo doped NiO NPs was increased as shown in Fig. 10. These findings showed that the antibacterial potential of the NiO NPs and Mo doped NiO NPs operated via generation of ROS against *S. aureus*, *B. subtilis*, gram-positive *P. aeruginosa*, and *E. coli*. No fluorescence was seen in the control (without nanoparticles), which shows that ROS was not generated in the strains in the absence of NPs. The fluorescence images show high fluorescent intensities at all the tested concentrations of NiO NPs and Mo doped NiO NPs indicated that the ROS generation was observed at all levels. The formed ROS interact with the cytoplasmic membrane, peptidoglycan layer, DNA, lipids, proteins, and other physiological processes of the bacterial strains and cause lysis of the cell. Additionally, the positively charged nickel molecules also react with the negatively charged microbial cell membrane and cause the proteins and other intercellular components to leak out and finally destroy the cell^35^.

**Figure 11:**
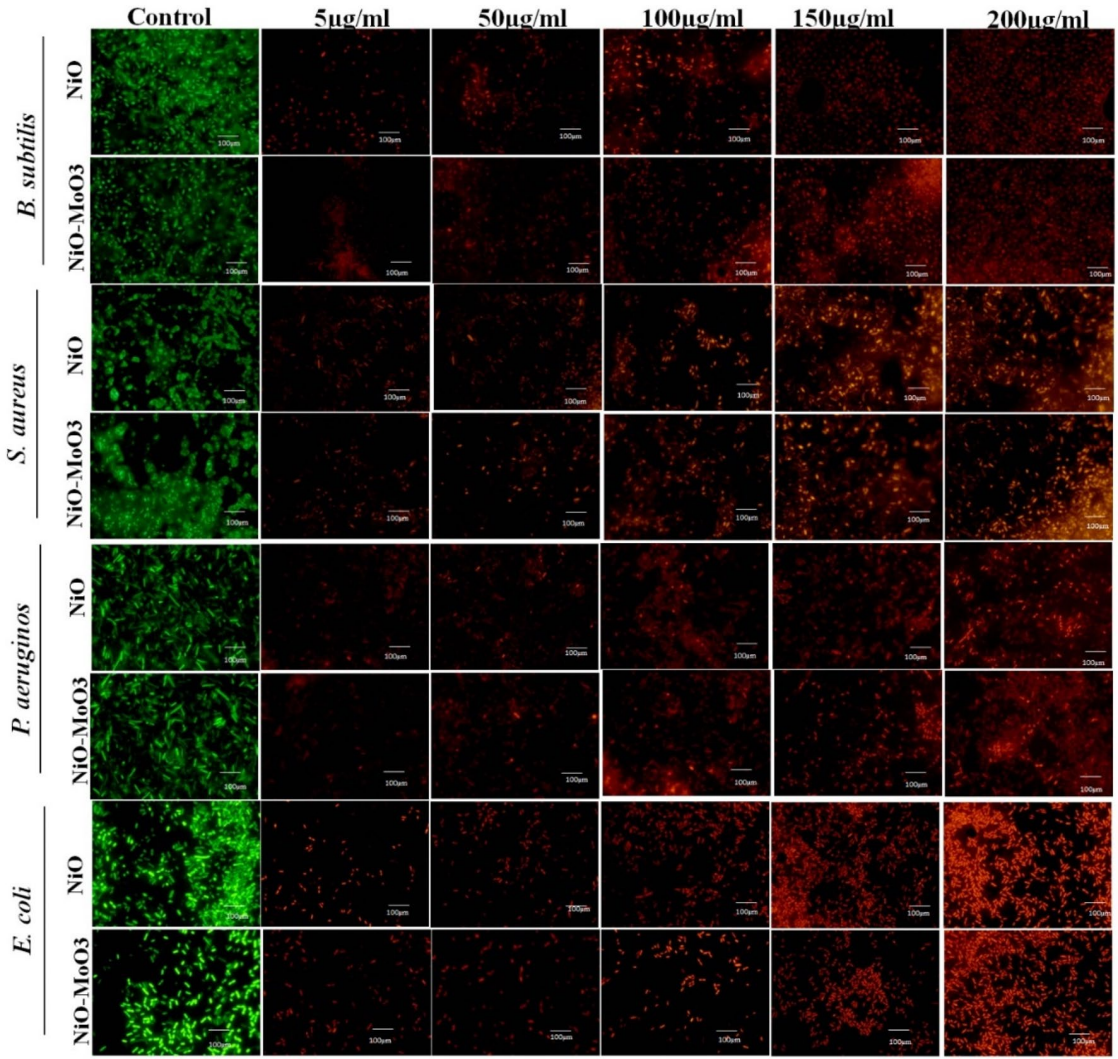
Fluorescence images depicts the live (green colour) and dead (red colour) bacterial cells *B. subtilis*, *S. aureus* gram-positive *P. aeruginosa*, and *E. coli*.

When bacteria were exposed to different concentrations of NiO NPs and Mo doped NiO, the morphology of the bacteria was observed by staining both living and dead cells with acridine orange (AO) and propidium iodide (PI). This should be mentioned that the cells with damaged or broken cell membranes can only be penetrated by PI. The green fluorescence was seen in the control, indicating that the bacterial cells were still viable. At higher concentrations (150 and 200 µg/mL) of NiO NPs and Mo doped NiO NPs, the treated bacterial cells revealed red colour (dead) confirming that the strong bactericidal activity was observed in case of the synthesized NPs (Fig. 11). The increase in the red color with the increase in the concentration of the NPs is indicative of the fact that the bactericidal activity of the NPs is concentration dependent phenomenon and it increases with the increase in concentration of the NPs^37^..

**Figure 12:**
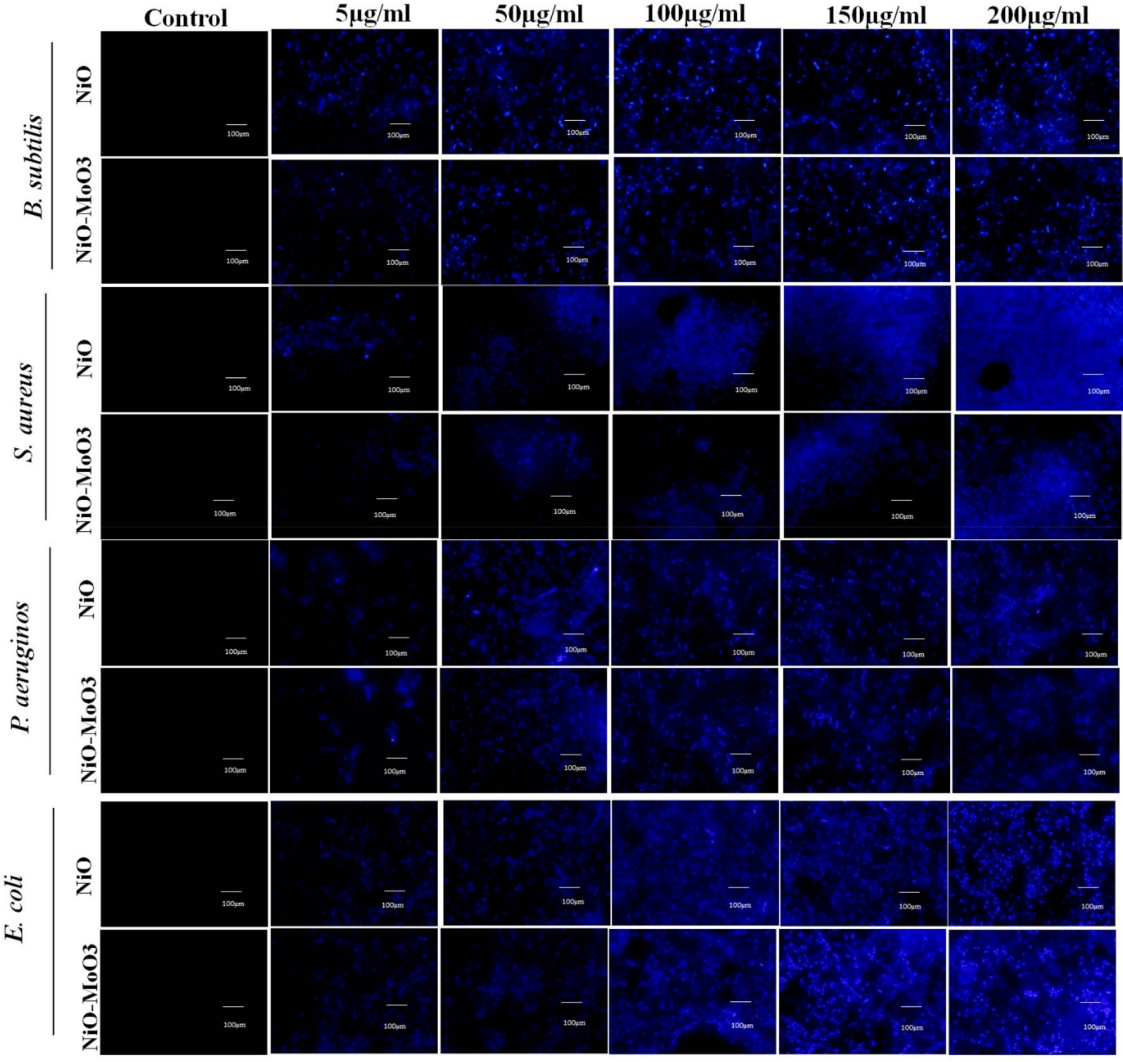
Fluorescence images represented the DAPI staining of bacteria which indicated the increased DNA damage with respect to increase the concentration of NiO NPs and Mo doped NiO ranges from 5 to 200 µg/mL.

The nuclear staining of bacterial cells with the 4',6-diamidino-2-phenylindole (DAPI) revealed the evidence for the DNA damage as shown in Fig. 12. The NiO NPs and Mo doped NiO NPs treated DAPI stained pathogenic bacteria displayed increased deep blue coloured spots when seen under fluorescent microscope. The blue coloration increases with the increase in the concentration of the NPs is indicative of the fact that the DNA damage was increased at the higher concentrations of NiO and Mo doped NiO NPs (100 and 200 µg/mL). The results demonstrated that the DNA damage is the concentration dependent parameter^38^.

**Figure 13:**
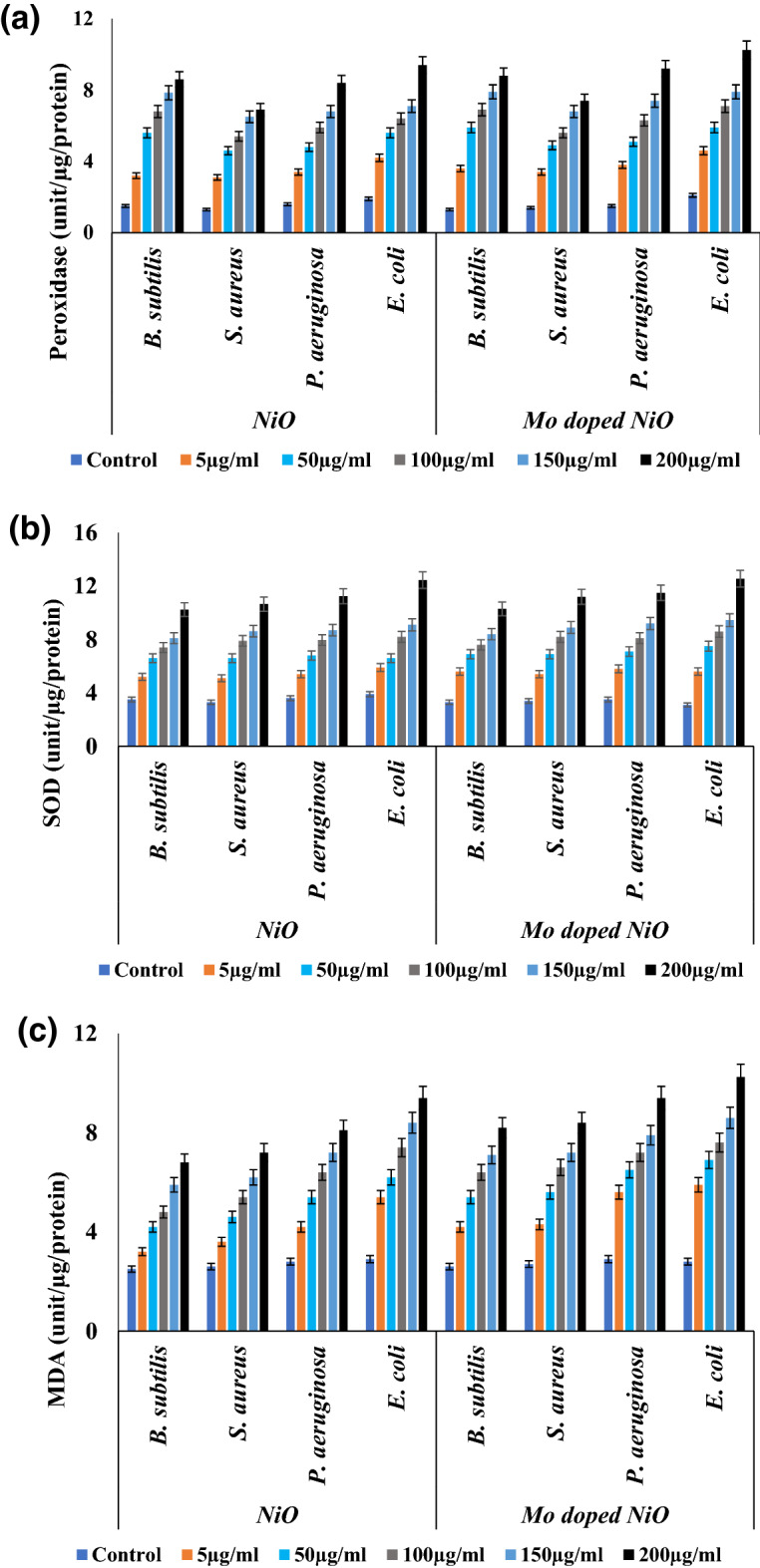
The NiO NPs and Mo doped NiO NPs treatment increased the levels of (**a**) POD, (**b**) SOD, (**c**) MDA assay. Data are presented as mean ± SD. **p < 0.05, *p < 0.01—statistically significant, ns-not significant.

By observing the activity of ROS-associated enzymes such as peroxidase (POD) and superoxide dismutase (SOD), the role of ROS generation and its consequences on the antioxidant system were assessed. The results showed that the bacterial cells exposed with the NiO NPs and Mo doped NiO NPs expressed enhanced levels of SOD and POD enzymatic activity. The POD and SOD activity was found to gradually enhance with the increase in the concentrations of NiO NPs and Mo doped NiO NPs from 5 µg/mL to 200 µg/mL as shown in Fig. 13A, B. Through measuring the MDA content, the membrane damage associated with the bacterial cells were assessed. When the bacterial pathogens were exposed to NiO NPs and Mo doped NiO, they often displayed an increase in the MDA concentration (Fig. 13C) which is indicative of the fact the ROS concentration in the system increases with the exposure of the cells with the NPs. Different ROS scavenging enzymes found in bacteria combat the ROS produced in non-stress conditions. However, in the case of overproduction of the ROS in the presence of NiO NPs and Mo doped NiO NPs at 200 µg/mL, the production of these ROS mediating enzymes also increases as well. The antibacterial mechanism relies on the generation of ROS, which causes DNA damage and eventually eliminates of the bacteria. This outcome is consistent with the prior report^39^. To conclude, the concentration of associated enzymes like SOD, and POD was found to increase with the increase in the concentration of NPs in the system as the exposure of the cells to the NMs enhances the ROS in the cell. Further tests revealed that the overproduction of ROS caused the damage to the cell membrane and adversely affected the DNA leading towards the lysis of the cell.

## Materials and methods

### Materials

The metal precursors such as nickel nitrate hexahydrate [Ni(NO_3_)_2_.6H_2_O; (99.99%)], sodium molybdate dihydrate [Na_2_MoO_4_.2H_2_O; (99.5%)], sodium hydroxide [NaOH; (97%)], and Urea [CO(NH_2_)_2_; (99.5%)] were purchased from sigma Aldrich. All the chemicals were of analytical grade and used without further purification. Deionized water was used as a solvent throughout the synthesis of the material. Acridine orange (AO), 2,2-diphenyl-1-picryl-hydrazyl-hydrate (DPPH), Hydrogen peroxide (H_2_O_2_), 2',7'-dichlorodihydrofluorescein diacetate (H_2_DCFDA), 2,3,5-triphenyl tetrazolium chloride (TTC), Propidium Iodide (PI), 4',6-diamidino-2-phenylindole (DAPI), potassium persulfate, trichloroacetic acid and other reagents and chemicals were purchased from HiMedia Laboratories Pvt. Ltd., (Mumbai, India). The pathogenic bacterial strains of *Bacillus subtilis, Staphylococcus aureus, Pseudomonas aeruginosa* and *Escherichia coli* were obtained from the Microbial Type Culture Collection (MTCC), Chandigarh, India.

### Methods

#### Synthesis of NiO NPs

In the typical synthesis, 2 mM Ni(NO_3_)_2_.6H_2_O solution was dissolved in 30 mL double distilled water and was further stirred for 15 min to attain the homogeneous solution. To the above solution, NaOH (precipitating agent) solution was added drop wise till the mother liquor attains pH 10 and stirring was further continued for 30 min. Then 1 mM of urea was dissolved in double distilled water and added to the above reaction mixture and stirred for next 2 h. The green colour precipitates were obtained and then, the reaction container was kept in an ultrasonicator for 3 h. The dispersed green precipitates were washed several times with the solution containing water and ethanol in 3:1 ratio to remove the unreacted precursor material and foreign materials present in the product. The obtained product was dried out at 80 °C and calcined at 600 °C for 5 h. The synthesized NiO NPs were stored in an air tight container for further analysis and application purposes^40^.

#### Synthesis of Mo doped NiO NPs

Briefly, 2 mM nickel nitrate precursor was dissolved in 30 mL of double distilled water and was stirred for 15 min. Then, 2 mM sodium molybdate was dissolved in the 30 mL double distilled water and was added to the nickel nitrate solution. The reaction mixture was constantly stirred for 1 h. To the above reaction mixture, 1 mM of urea was added and stirred for next 2 h. The pale green colour precipitates were obtained and the reaction medium was kept in an ultrasonicator up to 3 h. The dispersed precipitates were washed several times with the solution containing water and ethanol in the 3:1 ratio to eliminate the unreacted precursor material and foreign materials present in the product. The obtained product was dried out at 80 °C and calcined at 600 °C for 5 h. Finally, the synthesized Mo doped NiO NPs were stored in an air tight container for further analysis and application purposes^41^.

#### Characterization

The phase, structure, and average crystalline size of the synthesized NPs were found out by XRD analysis by using XPERT-PRO diffractometer with Cu Kα as a radiation source (λ = 1.540 Å, 40 kV, 15 mA) and it was recorded in the range of 10–90°. The average crystallite size is calculated using Debye Scherrer formula (D = Kλ/βcosθ), where K is the coefficient which have the constant value 0.89, β is full width half maximum, λ is X-ray wavelength. The morphology and average particle size of the material were analyzed by SEM using Bruker with EDX at an operating voltage of 15 kV. The atomic and weight percentages of the constituents was found by EDX analysis. The HR-TEM analysis was performed by JEOL-2100. The optical property was investigated by UV–Vis analysis using a JASCO spectrophotometer and recorded in the range of 200–800 nm with a scanning speed of 1000 nm/min. The FT-IR analysis was done with JASCO FT-IR spectrometer by using KBr as a standard to confirm the formation of metal oxide as well as presence of various functional groups present in the material.

### Antioxidant

#### DPPH assay

The DPPH (2,2-diphenyl-1-picryl-hydrazyl-hydrate) free-radical scavenging action of Ni and Mo doped NiO were determined as reported^17^. Briefly, 0.2 mM DPPH was added in methanol and different concentration of Ni and Mo doped NiO (0, 10, 50, 100 and 200 µg/mL) were prepared in methanol. The 50 µL of DPPH solution was added to microtiter plates containing various concentrations of NPs. Then, the reaction mixture was shaken and incubated in dark conditions for 30 min. The absorbance was measured at 570 nm and scavenging activity was calculated as follows:

$$\% {\text{ DPPH scavenging activity }} = \, \left( {{\text{Abs}}_{{{\text{control}}}} - {\text{ Abs}}_{{{\text{test}}}} /{\text{Abs}}_{{{\text{control}}}} } \right) \, \times {1}00$$

#### *ABTS*^*.*+^*scavenging assay*

Using distilled water, 2 mM of ABTS^**.**+^ was combined with 2.45 mM of potassium persulfate and left in the dark for 6 h at room temperature. After that, ABTS^**.**+^ was added to 0.1 mM of sodium phosphate buffer (KH_2_PO_4_). Following the mixing of 3 mL of NiO and Mo doped NiO NPs with 1 mL of ABTS^**.**+^ solution, the ABTS activity was calculated using the formula below

$${\text{ABTS}}^{ + } {\text{scavenging activity }} = \left[ {\left( {{\text{A}}_{{1}} - {\text{A}}_{{2}} } \right)/{\text{A}}_{{1}} } \right] \times {1}00$$

where A_1_ is the initial concentration of ABTS^**.**+^ , A_2_ is the final concentration of ABTS^**.**+^ after NPs treatment^42^.

#### Reducing power activity

Different concentrations (5 µg/mL, 50 µg/mL, 100 µg/mL, 150 µg/mL, and 200 µg/mL) of NiO and Mo doped NiO were treated with 200 mM sodium phosphate buffer and ferricyanide to measure the reducing power potential of NiO and Mo doped NiO NPs. This was followed by incubation at 50 °C for 20 min in the dark. The process was then halted by adding 50 µL of trichloroacetic acid. To get the supernatant, the reaction mixture was centrifuged at 3000 rpm for 10 min. 0.1% of ferric chloride was added to microtiter plates with 50 mL of supernatant, which were then incubated for 10 min. The absorbance at 700 nm was examined, and a high absorption indicated that NPs had a strong reducing power^43^.

#### Hydrogen peroxide scavenging assay

The effect of NPs to scavenging the hydrogen peroxide is studied the following method^20^. 40 mM of hydrogen peroxide was prepared using PBS. Then, as previously described, different concentrations of NiO and Mo doped NiO were treated with 0.6 mL of 40 mM H_2_O_2_ and incubated for 10 min before the measurement of absorbance at 230 nm^43^.

#### Superoxide radical scavenging activity of Ni and NM NPs

NiO and Mo doped NiO NPs were tested for their ability to scavenge superoxide radicals using EDTA (1 mM), 50 mM of PB (Phosphate buffer), and 0.02 mM of riboflavin. NBT (0.75 mM) was subsequently added to this solution. This reaction mixture was treated with NPs at concentrations ranging from 5 µg/mL to 200 µg/mL. After being exposed to fluorescent light for 7 min, the scavenging activity was measured at 560 nm^44^.

#### FRAP assay

The FRAP, according to^22^, was performed. To 0.2 M (1 mL) sodium phosphate, 1% potassium ferricyanide (1 mL) was added. Then, this mixture was added to 1 mL of NiO and Mo doped NiO NPs at varied concentrations (5 µg/mL, 50 µg/mL, 100 µg/mL, 150 µg/mL and 200 µg/mL) and it was incubated for 20 min at 50 °C. It was centrifuged at 3000 rpm for 10 min after 2.5 mL of 10% trichloroacetic acid was added to it. After 10 min of incubation, the supernatant (1.5 mL) was combined with distilled water (1.5 mL), 0.1% FeCl_3_, and 0.1 mL. The absorbance was then recorded at 700 nm^45^.

### Statistical analysis

The assays were performed in triplicates and data are presented as mean ± standard deviation. One-way analysis of variance (ANOVA) was followed to determine statistical differences between NiO NPs and Mo doped NiO treated and control groups, **p < 0.05, *p < 0.01 are statistically significant, ns-not significant.

### Antibacterial

#### Determination of antibacterial action

Antibacterial potential of NiO and Mo doped NiO NPs were evaluated using agar well diffusion method as described^23^ against gram positive (*S. aureus* and *B. subtilis*) and gram negative (*P. aeruginosa* and *E. coli*) bacteria. The bacterial strains were overnight cultured and spread on Muller Hinton Agar. Afterwards, wells were made with the help of metal corkborer and 100 µL of NiO and Mo doped NiO NPs at 1 mg/mL were added to respective wells. As a reference, streptomycin was used. Then the plates were incubated at 37 °C. The zone of inhibition (ZIC) was measured in millimetre. The experiment was conducted in triplicate.

#### Bacterial growth curve analysis

Bacterial growth kinetics assay was used to assess the bactericidal activity of NiO and Mo doped NiO NPs as reported^46^ with minor modifications. Various concentrations of NiO and Mo doped NiO NPs (5 µg/mL, 50 µg/mL, 100 µg/mL, 150 µg/mL, and 200 µg/mL) were loaded into 96 well plates and 10 µL of overnight grown bacterial cultures were added to each well. The plates were incubated at 37 °C for 20 h. The optical density measurement from each well was taken after every 2 h using microplate reader at 600 nm. Experiments were performed in triplicate and growth curves were plotted between optical density and time.

#### MIC and MBC

MIC and MBC against pathogenic bacteria was evaluated by broth microdilution method according to^46^. The NiO and Mo doped NiO NPs solutions were prepared at a concentration of 100 µg/mL. Nutrient broth (100 µL) was added to each well of microtiter plates followed by the addition of serially diluted NPs. Afterwards, 10 µL of bacterial inoculum was added into each well and incubated at 37 °C for 24 h. The absorbance was measured at 620 nm using microplate reader. TTC (2,3,5-triphenyl tetrazolium chloride) 10 µL was added followed by 30 min incubation period. Lowest concentration of nanoparticles that inhibit the growth of pathogenic bacteria is taken as MIC and compared to control. The experiments were performed with three replications. MBC was performed by diluting the MIC culture and subculture on sterile MH agar plates. Viable colony count method was followed to calculate the MBC.

#### Live/dead cell assay

In order to discriminate between live and dead bacteria, we followed^47^ approach with minor modification. In brief, 50 µL of various concentrations of NiO and Mo doped NiO NPs (5 µg/mL, 50 µg/mL, 100 µg/mL, 150 µg/mL, and 200 µg/mL) were treated with 100 µL of the pathogenic bacterial strains (1.5 × 10^8^ CFU mL^-1^) and incubated for 30 min at 37 °C. Nanoparticles untreated bacterial cells were used as a control. The reaction mixture was then centrifuged for 5 min at 5000 rpm, and the pellet was washed using PBS (Phosphate Buffer Saline). The AO and PI were employed in a 1:1 ratio to stain the pellets, which were then incubated for 30 min. The samples were washed with PBS to remove extra stain after 30 min of incubation, and examined by using a fluorescence microscope with the proper excitation and emission filters (430–470 nm) for these two dyes.

#### Intracellular ROS measurement

The ROS in bacterial cells were evaluated using the fluorescent probe H_2_DCF-DA according to^48^. The bacterial cultures (1 × 10^6^ CFU/ml) were exposed for 2 h to 100 µL of various concentrations of NiO NPs and Mo doped NiO NPs. The bacterial samples were then washed and suspended in PBS. Then, exposed to 100 µL of 1 mM H_2_DCFDA solution, and then left to incubate for 30 min at 37 °C in the dark. The bacteria were then lysed using lysis buffer, centrifuged for 5 min at 3000 rpm, and the supernatant's absorbance was determined spectroscopically at 520 nm. The experiment was performed three times. The fluorescent images of the NPs treated and control groups were measured using an upright fluorescence microscope, and the ROS level was recorded using a spectrofluorometer with excitation/emission filters 488/525 nm.

#### DNA damage assay

DAPI was used to analyse the DNA damage in bacteria. The selected bacterial pathogens were subjected to various concentrations of NiO and Mo doped NiO NPs for 96 h. The treatment and control were stained with DAPI (1 µg/ml DAPI in ethanol) at 37 °C for 15 min. After being washed twice with PBS, the stained bacterial cells were air dried. A fluorescence microscope (BX41, Olympus, Japan) was used to study the bacterial cells (Using a DAPI filter)^49^.

#### Biochemical assays

The bacterial extract was prepared using nutrient broth (5 mL), which included 10^8^ CFU/mL of *S. aureus, B. subtilis, P. aeruginosa, and E. coli*. The bacteria were exposed to NiO and Mo doped NiO at various concentrations and then, were cultured for a day. To obtain the crude enzyme extract, the bacterial cells were centrifuged at 7000 g for 10 min. The pellet was then sonicated. Bacteria that hadn't been exposed to NPs were used as controls. The POD activity, according to^50^, was measured in triplicate. The reaction mixture for POD analysis was prepared by mixing 1 mL of pyrogallol (0.01 M), 2 mL of 0.1 M PBS (pH 6.0), and 1 mL of 5 mM H_2_O_2_. Then, 1 mL of enzyme extract was incubated at room temperature for 5 min. The reaction was then stopped with 1 mL of H_2_SO_4_ (2.5 N), and the amount of purpurogallin produced was determined using a microplate reader plate reader at 420 nm. The SOD activity was assessed by utilizing the protocol suggested by^51^. The reaction solution was comprised of the following substances: 13.0 mM methionine, 6.3 mM NBT, 0.10 mM EDTA, 6.5 mM riboflavin, and PBS (pH 7.8). 500 µL of enzyme extract and 1.5 mL of the reaction solution. The mixture is then heated to 30 °C and left there for 10 min. After being placed in the dark, the absorbance was then recorded using an Eliza plate reader at 560 nm. The thiobarbituric acid was employed to quantify membrane damage by identifying the production of malondialdehyde (MDA) according to^50^. After being hydrated in 2.5% (w/v) trichloroacetic acid, 1 mL of enzyme extract was centrifuged at 12,000 g for 20 min at 4 °C. Thiobarbituric acid (0.5% (w/v)) reagent was produced in 20% (w/v) trichloroacetic acid, and 100 µL of the supernatant was added. Before being centrifuged at 12,000 g for 10 min at 4 °C, the reaction solution was first heated to 100 °C in a water bath. The absorbance was then measured at 532 nm using a microplate reader.

## Conclusion

Mo-doped NiO oxide nanoparticles were successfully synthesized, characterized, and investigated for antibacterial and antioxidant activity. The XRD analysis revealed that the pure NiO NPs had rhombohedral crystal phase while the Mo-doped NiO NPs exhibited monoclinic crystal phase. According to HR-TEM analysis, the pure NiO also revealed to have an irregular spherical shape morphology with an average particle size of 93 ± 4 nm, while Mo-doped NiO NPs exhibited a spherical morphology with an average particle size of 85 ± 3 nm. The pure NiO NPs and Mo-doped NiO NPs exhibited an absorption maximum at 365 and 349 nm, respectively, in the visible region. The NiO NPs and Mo doped NiO NPs were analysed for their ability to scavenge free radicals in numerous biochemical assays including DPPH, ABTS^**.**+^, FRAP, H_2_O_2_, reducing power assay, and SOD assays. All these assays revealed that the doped NiO NPs exhibited better results in comparison to the pure NiO NPs owing to their smaller size. Furthermore, the antioxidant potential of the NPs was found to be concentration dependent application. . Using the agar well diffusion method, the antibacterial potential of NiO and Mo-doped NiO NPs against gram-positive (*S. aureus* and *B. subtilis*) and gram-negative (*P. aeruginosa* and *E. coli*) bacterial strain was determined. It was again found out that the Mo doped NiO NPs exhibited better inhibition and lysis potential in comparison to the pure NiO NPs due to high penetration potential of doped NPs because of their small size. The mechanism associated with the bactericidal application of the NPs was also investigated which revealed that the exposure of the cell to the NPs enhanced ROS stress in the cell. Consequently, the concentration of associated enzymes like SOD, and POD was found to increase with the increase in the concentration of NPs in the system. Further tests revealed that the overproduction of ROS caused the damage to the cell membrane and adversely affected the DNA leading towards the lysis of the cell. All these findings suggest that the NiO and Mo doped NiO NPs are excellent materials for biomedical applications.

## Data Availability

The datasets used and/or analysed during the current study available from the corresponding author on reasonable request.
